# The clinical safety of disabled patients: Proposal for a methodology for analysis of health care risks and specific measures for improvement

**DOI:** 10.4317/medoral.18262

**Published:** 2013-02-05

**Authors:** Bernardo Perea-Pérez, Elena Labajo-González, Manuel Bratos-Murillo, Andrés Santiago-Sáez, Elena Albarrán-Juan, Alfonso Villa-Vigil

**Affiliations:** 1 Director of the Spanish Observatory for Dental Patient Safety (OESPO); 2 Member of the Spanish Observatory for Dental Patient Safety (OESPO); 3 Professor of the School of Legal and Forensic Medicine of Madrid. School of Medicine. Universidad Complutense de Madrid; 4 Founding President of the Spanish Society of Dentistry for the Disabled and Special Patients (SEOEME); 5 President of the General Dentists’ Council of Spain

## Abstract

The clinical risks associated with health care have been a known factor since ancient times, and their prevention has constituted one of the foundations of health care. However, concern for the risks involved in health care treatments has risen very significantly in recent years, becoming a modern current of concern for clinical health care risks which is referred to by the name of “patient safety” in the scientific literature. Unfortunately, there are no studies on patient safety in dental practice or case studies of adverse events in this practice. In addition to the lack of studies on adverse events in regular dental practice, there are even fewer references to treatment for disabled patients. In this article, we provide a “proposal for analysis” of the clinical risks associated with treating disabled patients, which will make it possible to evaluate the health care risks associated with the treatment of patients who have a specific disability, at one determined moment and in one specific environment.

** Key words:**Patient safety, disabled patients, health care risk.

## Introduction

The clinical risks associated with health care have been a known factor since ancient times. In fact, their prevention has constituted one of the foundations of health care, as stated in the Hippocratic oath, “Primum non nocere.”

Nevertheless, the concern about health care treatment risks has increased very notably in recent years, above all as a result of the publication of the book “To err is human: building a safer health system ” by the United States Institute of Medicine in the year of 1999. This study estimated that, in the United States alone, between 44,000 and 98,000 people die every year due to adverse events related with health care. Since the publication of the aforementioned book, the number of institutions interested in this topic and studies published about it have undergone a spectacular increase. Most notable due to their importance are the initiatives by the World Health Organization (WHO) through the “World Alliance for Patient Safety” (1-2).

Within the ontological realm, the initiatives arose much later, but at present the World Dental Federation (FDI) and the corporate entities in different countries are working on this topic (3-4). Very noteworthy is the creation in Spain of the Spanish Observatory for Dental Patient Safety (OESPO) under the auspices of the General Council of Odontologists and Stomatologists of Spain, the first institution dedicated to dental patient safety in the world. Moreover, the General Council of Odontologists and Stomatologists of Spain, at the request of the Observatory, approved a “Plan for Managing Clinical Risks in Dentistry” (5).

This modern current of concern for clinical health care risks is given the name of “patient safety” in the scientific literature. And this entire culture of “patient safety” revolves around the concept of the “adverse event” (6-8). An “adverse event” is some sort of harm produced to a patient as a result of the treatment itself, regardless of the underlying pathology. Adverse events may basically be of three types: errors, accidents or complications (9-10). In turn, they may be defined as avoidable or unavoidable. The ultimate objective of the whole patient safety culture is to avoid, to the greatest extent possible, the occurrence of avoidable adverse events and to detect unavoidable adverse events early on (so as to limit the damage they cause).

Unfortunately there are no sets of case studies on adverse events in dental practice. It is very difficult to obtain reliable data due to the dispersion of sources and the secrecy which tends to surround these types of incidents. And while there are not sufficient data regarding adverse events in regular dentist practice, there are even less referring to the treatment of disabled patients.

Common sense alone dictates that the health care risks for a patient with a physical, mental or sensorial disability must necessarily be greater. This is due to the fact that the risks caused by the treatment itself are added to the risks caused by the patient’s disability (11-12).

Those physical disabilities associated with walking-related problems lead to an increased risk of falling, above all if there are physical obstacles or slippery flooring at the dental office (13). On some occasions, this type of disability can complicate dental care and increase the risk of adverse events, as is the case with the risk of a patient falling while being transferred from a wheelchair to a dentist’s chair.

The various types of sensorial disabilities entail different risks (14). Visual disabilities lead to a great risk of falling or hitting physical obstacles at the dentist’s office. This type of disability can also increase the risk that, in the event that the dentist makes a maneuver “unexpected” by the patient, the patient may make some sudden movement that can cause some type of injury (cuts, burns, etc.). Visual limitations also increase the risk of confusion in drug treatments to be taken at home. Hearing disabilities, due to interference with the normal communication between patient and dentist, also increase the risk that the patient will not follow the dentist’s instructions (in the operative or post-operative stages) and that some harm may occur. Mental or psychological disabilities can limit a patient’s ability to understand, as well as decrease their control of impulses, making clinical practice much more difficult (15). They also create legal problems involving the patient’s ability to reach decisions.

In this article, we provide a “proposal for analysis” of the clinical risks associated with the treatment of disabled patients. This “proposal for analysis” will not provide any information which an experienced professional is not already quite familiar with. This type of analysis, very frequent in “patient safety,” attempts to systematize the study of health care risks so that none go unnoticed and in order to organize them in accordance with the level of danger they represent to patients. It is obvious that knowing these risk levels will help us organize the measures of prevention against them. At the same time, dentists with less experience will benefit from the greater experience of other professionals by using this methodology.

As a result of the application of the aforementioned methodology to general dental practice, in this article we also make a proposal of simple, specific measures which increase “patient safety” in accordance with the patient’s type of disability.

The objective of this article is to propose a methodology which allows us to evaluate the health care risks associated with the treatment of a patient who has a specific disability, at a specific time and in a specific environment. The use of this methodology must keep certain risks from not being taken into consideration, and we repeat once again that accurate knowledge of these risks is necessary for their proper prevention.

## Analysis of care-related risks among disabled patients ( Table 1)

-Analysis of care-related risks associated with patients and the characteristics of their disability.

**Table 1:**
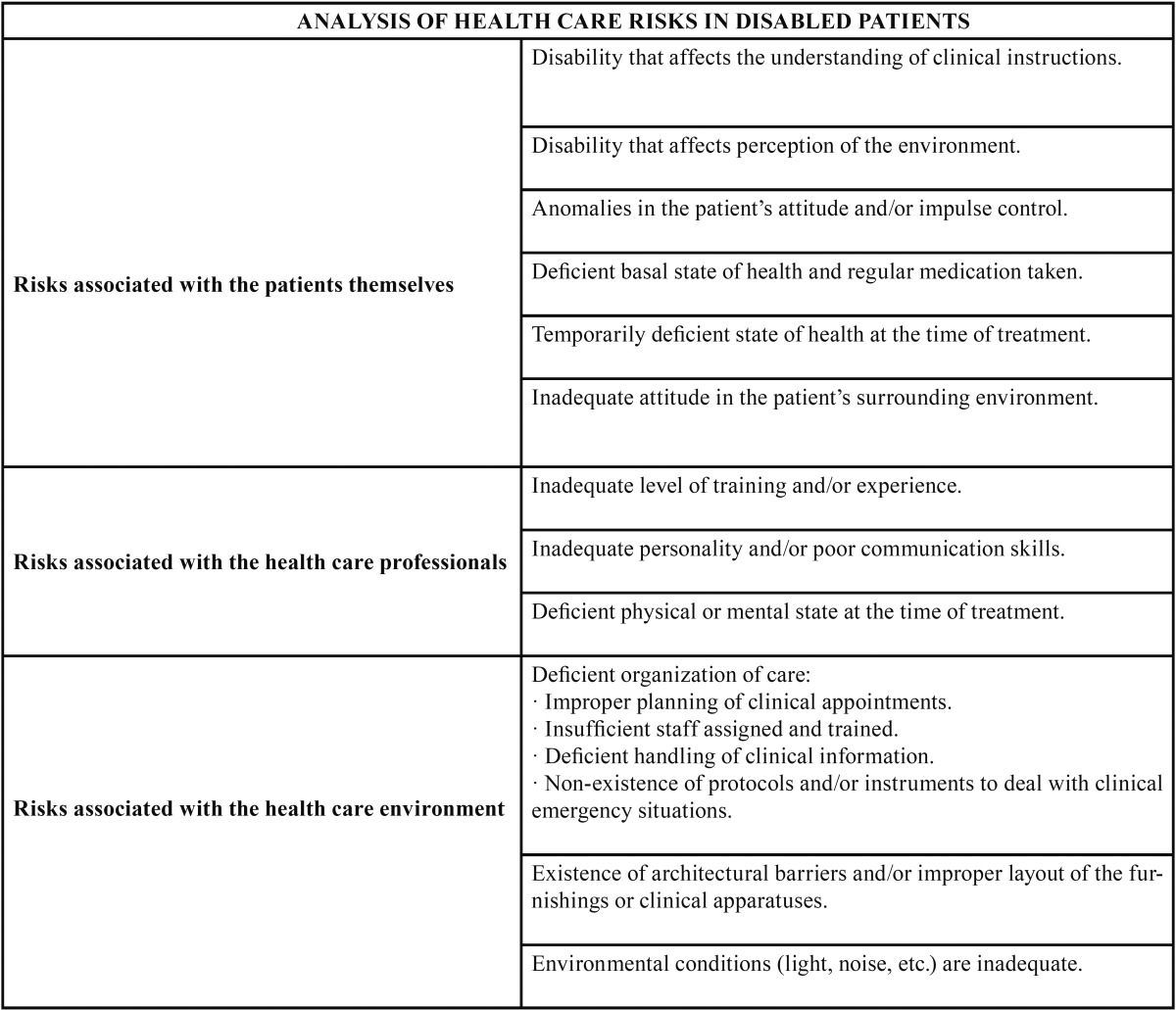
Analysis of health care risks among disabled patients.

The risks specifically associated with patients’ disabilities are as diverse as the types of disabilities they have. The clinical problems entailed by a motor disability are completely different from those of a sensorial disability or mental disability (13). Nevertheless, many risks are shared by some or all of the categories of disabilities mentioned above.

As a prior step before analyzing the health care risks related with the status of a disabled patient, we recommend performing a very careful description of the disability, its potential clinical repercussions and the pharmacological treatments associated with it. We also recommend a prior evaluation of the patient’s disability so as to adapt the way you communicate, and due to the important legal repercussions it may lead to.

The methodology for analysis of risks associated with the disabled patients themselves is as follows:

a. Risks associated with disorders involving understanding disabilities.

Disabilities involving understanding may translate into the following, in terms of effects on health care:

• Problems prior to treatment (accepting treatment, understanding therapy alternatives, validity of informed consent, etc.).

• Problems throughout the course of treatment (understanding instructions about position or immobility, etc.).

• Problems after treatment (understanding post-operatory instructions, need to follow the treatment, etc.).

b. Risks associated with the ability to perceive the surrounding environment.

These risks may translate into, for instance, an inability to see the physical obstacles at the health care center (with a greater likelihood of falling or hitting something), difficulties in hearing our verbal instructions, or the patient’s inability to see the maneuver that we are about to perform and anticipating the sensations it will produce.

c. Risks associated with patient attitude and impulse control.

In this category, we would include the patient’s level of cooperation and the potential for sudden unexpected movements while clinical maneuvers are being performed.

d. Risks associated with the patient’s basal state and regular medication.

Association of many disabilities is something frequent in organic syndromes. It is also frequent for these organic syndromes to lead to specific risks or taking drugs which may interact with those which we administer.

e. Risks associated with the patient’s state of health at the time of treatment.

In this section, we would include specific health disorders and not those related with the underlying disease. For example, any acute illness, significant states of stress or fatigue, or any other factor which may increase the potential that an adverse health care event may arise.

f. Risks associated with the attitude of the people close to the patient (family or personnel of the institutions where he or she re-sides).

Although this factor is not strictly related with the patients themselves, for the purposes of methodology we shall considered it to be so. Depending upon the patient’s level of dependence upon others, the role of the family (or the people who take care of the patient) may be fundamental. If the level of dependence is high, it is the family which must understand the therapeutic recommendations before and after treatment and ensure that they are complied with. From a legal perspective, as well, it is the family which must give consent to have the treatment performed.

-Analysis of the health care risks related with health care professionals.

In this section, we refer to all of the staff working on the health care team, and not just the dentist. It is obvious that many of the factors which can lead to adverse health care events arise from the auxiliary staff, which can sometimes spend more time in contact with the patient than the dentists themselves.

The methodology for analyzing the health care risks related with health care professionals is as follows:

a. Risks associated with the professional’s level of training and experience.

Amongst these risks, we would differentiate two types, those related with the specific treatment to be provided and those related with the treatment of disabled patients.

b. Risks associated with the professional’s personality and communication skills.

We would include within this section a wide variety of factors, from the professional’s empathy to his or her patience in the event of interruptions in treatment. Moreover, communication skills are more important when dealing with disabled patients than in regular clinical practice. On a frequent basis, professionals must communicate at almost the same time with the patient and his or her family, changing the form and contents of the messages, with all the difficulties that this creates.

c. Risks associated with the state of the professional at the time of treatment.

To be included in this section are all of the factors related with the dentist’s state of health (whether temporary or chronic) which may alter his or her level of professionalism. It also includes situations of fatigue or stress and psychological problems.

-Analysis of the clinical risks related with the surrounding health care environment.

The clinical risks related with the surrounding health care environment in which the treatment is provided are of many types and vary greatly from one center to the next.

The methodology for analysis of clinical risks related with the health care environment which we propose is as follows:

a. Risks associated with the organization of care.

The organization of care is one of the factors that creates the greatest number of “latent risks” in health care. A detailed analysis of these risks would go beyond the limits of the objectives of this article. However, we would like to emphasize a few points:

• Problems planning clinical appointments.

The most frequent is not taking into account that the treatment of disabled patients usually requires more time. This, in turn, tends to lead to excessive pressure on the health professionals in providing care.

• Problems related with the staffing to deal with this type of treatments.

Disabled patients usually require constant supervision, and therefore enough staff must be working to provide this care. Moreover, this staff must have the proper training and experience. These problems related with the amount and enablement of the staff mainly arise in situations when there are unforeseen absences of regular personnel.

• Problems related with the existence of proper handling and conveyance of clinical information.

Very frequently, disabled patients are unable to warn dentists about a problem which arises during treatment, either because they are unaware of the problem or because they cannot express it. Because of this, it is a critical need for the health care personnel to have all of the updated clinical information regarding this type of patients.

• Problems related with the appearance of clinical emergency situations.

Although no data are available, it seems reasonable to assume that this type of patients may suffer from clinical emergencies with a greater frequency than others. Therefore, it is essential to have protocols for action and the materials necessary to deal with this type of situations.

b. Risks associated with the existence of physical barriers in the dentist’s office.

In this section, we would include various risks: the existence of architectural barriers (stairs, ramps, etc.); an improper layout of furnishings and care apparatuses; and other miscellaneous factors such as the existence of slippery floorings, etc. We must always bear in mind that many types of disabilities include impairments in the perception of this type of barriers, or impairments in stability which lead to falls.

c. Risks associated with the center’s environmental conditions.

Here we are basically referring to the lighting and noise conditions, which can affect both disabled patients and dentists themselves. For example, a high sound level, or the occurrence of a sudden, unforeseen sound, may alter the state of the patient with a mental disability.

## Proposal for improvement of clinical safety in dental care for patients with disabilities

On the basis of the analysis of health care risks in general dental treatment, the following measures are proposed to improve safety for disabled patients. These recommendations must be known and practiced by both the dentist and any auxiliary staff.

-General Recommendations:

1. The dentist must introduce himself to the patient and determine for himself the patient’s ability and the limitations which the decreased ability may entail in terms of treatment. This evaluation must be corroborated through an interview with family mem-bers or other friends or related persons.

2. The patient must preferably come to the dentist’s office with accompaniment, though the patient must also be asked about the degree of confidentiality the dentist must maintain in terms of any accompanying persons.

3. Adapt communications to the patient’s ability to understand. This includes providing clear and accurate information (to the patients and/or friends and family) in understandable language.

4. Be especially careful when asking about the clinical background and about medication.

5. Adapt the characteristics of the care given to the treatment of this type of patients (training for personnel, duration of appointments, etc.).

6. Offer help, but do not force it on such patients.

7. Attempt to group treatments together to reduce the number of times the patient must go to the dentist’s office to a minimum.

8. Eliminate, to the greatest extent possible, any physical obstacles and architectural barriers.

9. Reduce noise pollution and factors which cause distraction.

10. Make sure that the patient and/or his friends and family have properly understood the information provided, using targeted questions (especially as regards taking medication or any maneuvers to be performed outside of the dentist’s office). Especially make sure that they are aware of the alarm signals or “red flags” to watch out for in terms of complications and how to act if any such symptoms occur (including the way in which they can contact the clinic).

11. Make sure that the patient is in proper condition for leaving the dentist’s office and that he is aware of the way to contact you in the event that any complication related with the treatment you have given comes about.

-Specific Recommendations.

-Sense-related Disabilities.

-Visual Disability.

a. Inform the patient of your name and role so that he is familiar with your voice.

b. Eliminate any physical obstacles and take the patient to the waiting room, as far as the dental chair, indicating the proper position and offering your assistance as much as the patient requests.

c. Strengthen communication with the patient in order to replace any visual limitations to the greatest extent possible. Inform the patient about what you will be doing at all times, and especially warn him if any tactile, painful or sound-related stimuli will be occurring which might surprise him.

d. Provide a warning when you will be leaving the room, and always leave the patient in contact with some sort of specific physical reference.

e. Inform the patient and provide a detailed description of the medications which you prescribe, attempting to bear in mind any possible confusions. Choose therapeutic formats which are different from the drugs which the patient already regularly takes.

-Hearing Disability.

a. Ask whether the patient knows how to read lips or uses sign language. If the patient uses sign language and is accompanied by someone who can act as an interpreter, situation this interpreter within the room in a location where the patient can see him or her easily.

b. In the event that you must raise your tone of voice in order for the patient to hear you, or if the patient reads your lips, you must speak slowly and use short, clear sentences. Make sure that you have been understood.

c. Provide information in a written form (clear and legible) to the greatest extent possible.

d. Attempt to avoid prescribing drugs which may damage the patient’s ability to hear (e.g., aminoglucoside antibiotics).

-Motor Disability.

a. Eliminate, to the greatest extent possible, any physical barriers which entail some danger or impediment that keeps these patients from moving about.

b. Accompany and help the patient, as much as requested, to the waiting room and the room where dental care is given.

c. If it is necessary to move the patient from a wheelchair to a dental chair, make sure the risk of falling is kept at a minimum.

d. Make sure that the patient can stand up from the dental chair or be moved into a wheelchair without feeling any symptoms that might cause the patient to fall.

-Mental and Intellectual Disability.

a. Evaluate the degree of mental disability to adapt your communication to the patient’s level of understanding.

b. If the patient’s ability level is low, locate his legal guardians, who must act as intermediaries (always informing the patient to the extent to which his or her ability allows).

c. Make sure that these intermediaries understand your instructions properly.

d. Bear in mind that patients with this type of disability may have greater difficulty controlling their behavior and anxiety. Act accordingly by attempting to convey a feeling of peace of mind, informing them in advance of any moves you will be making, and remaining on your guard about sudden movements.

e. The instructions about pre- or post-operatory care and medication must also be given to the guardians in writing.

f. The guardians must be clearly informed about any clinical alarm signals or “red flags” and the attitude they should acquire if any such red flags arise.

## Conclusions

Any dental treatment bears with it the potential for causing some sort of adverse health care event. This risk is clearly increased amongst patients who have some sort of disability (physical, mental or sensorial). Evaluating the care-related risks of these patients is an essential factor in being able to prevent their occurrence (or at least limiting their consequences). This risk evaluation must be systematic and thorough. It must also distinguish between the systemic risks inherent to care (those which are constant) and any specific risks (those which are due to circumstances limited in time).

The evaluation of care-related risks amongst disabled patients must focus on three factors: those related with the patients themselves, their disability type and their family environment; those related with the health care professionals who provide their care; and those related with the care-related environment.
